# Draft Genome Sequence of the Uropathogenic *Herbaspirillum* *frisingense* Strain* ureolyticus* VT-16-41

**DOI:** 10.1128/genomeA.00279-17

**Published:** 2017-04-27

**Authors:** Victor Tetz, George Tetz

**Affiliations:** Human Microbiology Institute, New York, New York, USA

## Abstract

*Herbaspirillum frisingense* strain* ureolyticus* VT-16-41 is a clinical cystitis isolate. Here, we report the draft genome sequence of the uropathogenic *H. frisingense* strain* ureolyticus* VT-16-41, which contains various antibiotic resistance genes and virulence factors that enable it to colonize and persist in the urinary tract.

## GENOME ANNOUNCEMENT

Urinary tract infections (UTIs) are considered among the most common bacterial diseases and are predominantly caused by members of the *Enterobacteriaceae* family (1). *Herbaspirillum* spp.—Gram-negative, motile bacteria belonging to the *Oxalobacteraceae* family—have only recently been considered potentially pathogenic and are now implicated in a number of human pathologies, such as cellulitis and bacteremia. These bacteria have been isolated from the respiratory tract, urine, eye, and ear samples (2–4). In contrast, *Herbaspirillum frisingense* has never been described as a human UTI pathogen (5). Using a developed workflow, combining microbiological and genetic processes, we have, for the first time, isolated* H. frisingense* from the bladder of a human patient with a UTI (6). The 16S rRNA gene of the isolate was sequenced and found to possess 99% identity with that of *H. frisingense*.

The whole genome was sequenced using Illumina HiSeq 2500 sequencing technology (Illumina GA IIx, Illumina, CA, USA), according to the manufacturer’s protocol. A draft genome was assembled using SPAdes version 3.5.0 with 143-fold average coverage (7). The assembled 283 contigs totaled 5,837,613 bp, and the G+C content was 62.0%. The assembled sequences were annotated using the NCBI Prokaryotic Genome Annotation Pipeline and the Rapid Annotations using Subsystems Technology (RAST) server (8, 9). The genome comprises 5,204 coding sequences, 56 tRNAs, 6 rRNAs, and 4 ncRNAs. An *in silico* DNA-DNA hybridization (DDH) analysis, using the Genome-to-Genome Distance Calculator (GGDC2.1) algorithm, produced a DDH value of 83.20%, which indicates that *H. frisingense* strain* ureolyticus* VT-16-41 is a new strain belonging to the species *H. frisingense* (5).

A genome analysis revealed the presence of numerous virulence factors that clearly contribute to the bacterium’s virulence in UTIs: hemolysin D; urease subunits alpha, beta, and gamma; type IV pilin; adhesins (sigma-fimbriae tip adhesion); and flagellar proteins (10). In addition, the genome was found to contain iron-acquisition systems that are known to be an important characteristic of uropathogenic bacteria, because the availability of iron is extremely restricted in the urinary tract: TonB-dependent siderophore receptor, ferroxidase, iron-sulfur cluster-binding protein, ferric iron ABC transporter, and iron-uptake factor PiuB (11). The genome analysis also identified antimicrobial resistance genes encoding resistance to metals (*CopD, ArsH*), bleomycin, RND, ABC, MFS, and MATE family efflux transporters. Further research of *H. frisingense* strain* ureolyticus* VT-16-41 will result in a better understanding of its implication in UTIs.

### Accession number(s).

This complete genome sequence has been deposited in NCBI/GenBank under the accession number MUXB00000000.
